# Anxiety and depression during the COVID-19 pandemic in Kuwait: the importance of physical activity

**DOI:** 10.1186/s43045-020-00065-6

**Published:** 2020-11-03

**Authors:** Khaled E. Alsharji

**Affiliations:** Department of Physical Education & Sports, College of Basic Education, Public Authority for Applied Education & Training, Kuwait City, Kuwait

**Keywords:** Anxiety, Depression, COVID-19, Physical activity, Physical inactivity

## Abstract

**Background:**

In Kuwait, as in much of the world, COVID-19 epidemic has been spreading since February 2020. The government of Kuwait has taken several measures to minimize virus spread. The epidemic and measures to contain it will likely psychologically influence individuals. This study investigated the prevalence of anxiety and depression symptoms in Kuwaiti nationals and expats. The study’s secondary aim was to identify the association between sociodemographic characteristics and physical activity (PA) engagement, with psychological disorders such as anxiety and depression during the COVID-19 pandemic in Kuwait. A two-section survey was used to examine sociodemographic characteristics and PA engagement, and generalized anxiety (GAD-7) and depression symptoms (PHQ-9).

**Results:**

Results of this study indicated that 53.7% and 59.6% of the respondents experienced anxiety and depression. The multiple regression models significantly predicted anxiety and depression (*P* < 0.001). In addition, PA, gender, education, and age were significantly associated with anxiety (*P* < .05), while PA, gender, age, and marital status were significantly associated with depression (*P* < .05).

**Conclusions:**

Findings suggest that the COVID-19 outbreak may have a greater psychological impact on women, younger individuals, married people, and individuals with a bachelor’s degree. However, regular PA appears to be an important immediate and long-term factor in reducing symptoms of anxiety and depression during an epidemic.

**Supplementary Information:**

The online version contains supplementary material available at 10.1186/s43045-020-00065-6.

## Background

The novel coronavirus 19 (COVID-19) first emerged in December 2019 in Wuhan, China [34]. Subsequently, it has spread fiercely around the world compelling the World Health Organization (WHO) on March 11, 2020, to declare the COVID-19 outbreak as a pandemic. The first confirmed case in Kuwait was announced on February 24, 2020, while the first death was reported on April 4, 2020. As of 12 August, there were 73,785 confirmed cases of COVID-19, with 65,451 recoveries and 489 deaths.

COVID-19 is the most recently discovered member of several coronaviruses with many similarities with severe acute respiratory syndrome (SARS) and Middle East respiratory syndrome (MERS). The virus is highly contagious and infectious affecting all segments of the population. However, the elderly and patients with preexisting conditions including diabetes and cardiovascular, respiratory, and immune diseases are more susceptible and experience more severe symptoms. The virus is transmitted via close contact (< 2.0 m), droplets (from coughing/sneezing), and in rare cases airborne. It attacks primarily the respiratory system causing a wide range of cold-like symptoms, most common are fever, cough, sneeze, runny nose, sore throat, chills, headache, loss of taste or smell, tiredness, muscle aches, and chest pain. However, in some cases vulnerable patients may experience shortness/difficulty of breathing and possible fatality.

After a rapid increase in cases, the Kuwaiti government implemented rigorous measures to intercept the virus spread. The Kuwaiti government suspended work, schools, universities, public events, social gathering, and closed boarders and public parks. Subsequently constraining people to home confinement and the entire country was lockdown. These measures can lead to social isolation and loneliness [26]. However, among the procedures, the government allowed a daily 2 h of grace period, during which many people may use this time to engage in physical activity (PA).

The combination of the virus complications, implemented governmental procedures, and the absence of pharmacological interventions, most likely people would experience emotional, psychological, and behavioral changes [6, 25]. Cao et al. [9] argued that when faced with highly contagious diseases people may experience unbearable psychological distress that may trigger harmful behaviors [23]. Subsequently, these feelings can lead to anxiety, depression, and poor mental health, and, in some cases, suicidal ideation [7, 26]. In fact, in a recent study, 53.8% reported moderate/severe psychological effect; 28.8% reported moderate/severe anxiety symptoms; 16.5% reported moderate/severe depressive symptoms; and 8.1% reported moderate/severe stress levels [31].

Regular participation in PA is frequently cited as the first step in lifestyle modifications to prevent and manage chronic disease [4], including psychological disorders. Regular participation in PA seems to improve self-efficacy [22], while reducing anxiety [17], depression [16], and negative moods [8, 11]. The global standard recommendations for PA—150 min/week of moderate PA or 75 min/week vigorous PA [33]—is effective in treating psychological disorders such as anxiety, depression, stress, and panic. Therefore, given these mental and psychological benefits, PA should be incorporated into epidemic risk reduction programs. Even during a quarantine, or total curfew, PA can be carried out at home [20].

At the time of this study, the COVID-19 outbreak curve indicated that Kuwait had approached the epidemic peak. Consequently, symptoms of psychological disorders (i.e., anxiety and depression) have likely already emerged [31]. PA, as indicated earlier, could be an important method for reducing these symptoms. Therefore, this study aimed to investigate the prevalence of generalized anxiety and depression symptoms in Kuwaiti nationals and expats. The study’s secondary aim was to identify the association between sociodemographic characteristics and PA engagement, with psychological disorders such as anxiety and depression during the COVID-19 pandemic in Kuwait. The author expects to increase prevalence of anxiety and depression during COVID-19 in Kuwait. Additionally, age, gender, marital status, and occupations would be associated with psychological status among the participants.

## Methods

### Participants and study design

A web-based cross-sectional survey was first disseminated via official emails to the Public Authority for Applied Education and Training (PAAET), the largest higher education entity in Kuwait and includes institutions from every governorate in the country. PAAET’s email list includes current/former students, teachers, trainers, faculty members, and current and retired employees. It therefore offers a representative microcosm of Kuwaiti society. The survey link was sent through social media (Twitter, Facebook, and Instagram). The survey was administered via the Google Form platform and was anonymous to ensure confidentiality and reliability of the data. Respondents were selected by snowball sampling, and each respondent was encouraged to forward the link to other family members, friends, and colleagues. The survey was conducted from 21 June to 11 July.

### Instruments

The survey is divided into two sections: (1) sociodemographic characteristics and PA engagement and (2) level of anxiety and depression. The sociodemographic characteristics and PA engagement were obtained with questions about gender (male or female), age, marital status (married or unmarried), occupation (employed, student, retired, or unemployed), and education level (high school or less, bachelor’s, or graduate degree). Additionally, in order to obtain PA engagement, the participants were asked to respond yes/no to two inquiries since the emergence of COVID-19. These inquiries were as follows: I do moderate (≥ 150 min/week) or vigorous physical activities (≥ 75 min/week). These responses were used to determine whether or not the respondents met the global PA standards [33].

The Patient Health Questionnaire (PHQ) is a self-administrated instrument with six modules measuring six disorders: depression (PHQ-9), generalized anxiety (GAD-7), panic, somatization, and eating and alcohol abuse disorders [28]. Respondents completed the Arabic versions of GAD-7 and PHQ-9 [2] to assess anxiety and depression, respectively. The GAD-7 has been translated to Arabic, found reliable (Cronbach’s alpha = 0.763), and used extensively in several Arab countries [2]. Respondents reported anxiety symptoms using a 4-item Likert scale ranging from 0 (not at all) to 3 (nearly every day). Seven questions assessed the frequency of anxiety symptoms over the past weeks with a total score ranged from 0 to 21 (normal = 0–4; mild = 5–9; moderate = 10–14; severe = 15–21). The questions inquired about feeling nervous, anxious, on edge, or worrying about different things; feeling restless so that it is hard to sit still; getting tired very easily; muscle tension, aches, or soreness; trouble falling asleep or staying asleep; trouble concentrating on things, such as reading a book or watching TV; and becoming easily annoyed or irritable [2, 28].

Similarly, the PHQ-9 has also been translated, found reliable (Cronbach’s alpha = 0.857), and used extensively in the Arab countries [2] and been found to be reliable. Respondents reported depression symptoms using a 4-item Likert scale ranging from 0 (not at all) to 3 (nearly every day). Nine questions assessed depression levels over the past weeks, with a total score ranged from 0 to 27 (i.e., normal = 0–4; mild = 5–9; moderate = 10–14; moderate to severe = 15–19; and severe = 20–27). The questions inquired about little interest or pleasure in doing things; feeling down, depressed, or hopeless; trouble falling or staying asleep, or sleeping too; feeling tired or having little energy; poor appetite or overeating; feeling bad about yourself—or that you are a failure or have let yourself or family down; trouble concentrating on things, such as reading the newspaper or watching TV; moving or speaking so slowly that other people could have or the opposite; and thoughts that you would be better off dead or of hurting [2, 28].

### Ethics approval and consent

This study was conducted in accordance with the Declaration of Helsinki and was approved by the ethics committee of the Ministry of Health Standing Committee for Health Research. Electronic informed consent was obtained from each respondent prior to starting the investigation. Participants could withdraw from the survey at any time without providing any reason.

### Statistical analysis

Descriptive analyses were applied to describe the sociodemographic characteristics, PA status, and levels of anxiety and depression among members of the Kuwaiti population during the COVID-19 outbreak. The data is presented in mean ± SD, frequency, or percentages. A chi-square test (χ^2^) was used to compare the differences in symptoms of anxiety and depression between groups. In addition, multiple regression models were performed to investigate the potential predictors for anxiety and depression symptoms during the COVID-19 outbreak in Kuwait. All data were analyzed using Statistical Package for Social Sciences (SPSS) version 25.0. A two-tailed *P* value < 0.05 was considered statistically significant.

## Results

### Sociodemographic characteristics and physical activity status

The sociodemographic characteristics and the PA status of the respondents are shown in Table 1. A total of 1284 participants were included in the analysis. Of the 1284, 55.1% were males, 96.6% Kuwaitis, 52.6% married, 59.1% holding a bachelor’s degree, 68.1% were students, and the mean (SD) age was 33.44 ± 11.09 years. Among all respondents, 53.9% were inactive, while 46.1% were physically active.

**Table 1:** Sociodemographic characteristics stratified by physical activity (PA) status during the outbreak of COVID-19 in Kuwait

Sociographic characteristics		*N* = 1284	Inactive	Active	χ^2^	*P* value
			693	591		
			53.9%	46.1%		
Gender	Male	707	366	341	3.08	0.079
		55.1%	51.8%	48.2%		
	Female	577	327	250		
		44.9%	56.7%	43%		
Marital Status	Unmarried	608	312	296	3.28	0.07
		47.4%	51.3%	48.7%		
	Married	676	381	295		
		52.6%	56.4%	43.6%		
Education	HS & Less	110	66	44	18.692	< 0.001
		8.6%	60.0%	40.0%		
	Diploma	165	100	65		
		12.9%	60.6%	39.4%		
	Bachelor’s	759	421	338		
		59.1%	55.5%	44.5%		
	MS or PhD	250	106	144		
		19.5%	42.4%	57.6%		
Occupation	Employer	286	154	132	7.498	0.058
		22.3%	53.8%	46.2%		
	Student	874	458	416		
		68.1%	52.4%	47.6%		
	Retired	67	45	22		
		5.2%	67.2%	32.8%		
	Unemployed	57	36	21		
		4.4%	63.2%	36.8%		

### Prevalence of anxiety

Table 2 shows that the anxiety score of 46.34% of respondents fell within normal range (GAD-7 = 0–4); 53.66% of respondents recorded scores indicative of anxiety (GAD-7 = 5–21). In addition, the data showed that moderate and vigorous PA during the COVID-19 outbreak was significantly associated (*P* < 0.001) with anxiety. There was significant difference in the prevalence of anxiety between physically active and physically inactive people; there was also a significant difference in the prevalence of anxiety by gender (*P* < 0.001), and the prevalence of anxiety was significantly higher among college students (*P* < 0.006).

**Table 2:** Prevalence of generalized anxiety disorder (GAD-7) among groups during the outbreak of COVID-19 in Kuwait

Sociodemographic characteristics		Levels of anxiety				***Anxious***	*x*^*2*^	*P* value
		Normal	Mild	Moderate	Severe			
		595	395	150	144	689		
		46.34%	30.76%	11.68%	11.21%	53.66%		
Physical activity	Physically inactive	287	211	92	103	406	29.068	< 0.001
		41.40%	30.40%	13.30%	14.90%	58.6%		
	Physically active	308	184	58	41	283		
		41.40%	30.40%	13.30%	14.90%	47.9%		
Gender	Female	224	172	84	97	353	49.772	< 0.001
		38.80%	29.80%	14.60%	16.80%	61.2%		
	Male	371	223	66	47	336		
		52.50%	31.50%	9.30%	6.60%	47.5%		
Education	High school and less	49	32	13	16	61	22.968	0.006
		44.50%	29.10%	11.80%	14.50%	55.5%		
	Diploma	64	46	34	21	101		
		38.80%	27.90%	20.60%	12.70%	61.2%		
	Bachelor’s degree	351	244	76	88	408		
		46.20%	32.10%	10.00%	11.60%	53.8%		
	Higher education	131	73	27	19	119		
		52.40%	29.20%	10.80%	7.60%	47.6%		
Marital status	Single	261	204	72	71	347	6.068	0.108
		42.90%	33.60%	11.80%	11.70%	57.1%		
	Married	334	191	78	73	342		
		49.40%	28.30%	11.50%	10.80%	50.6%		
Occupation	Employer	126	88	36	36	160	15.884	0.069
		44.10%	30.80%	12.60%	12.60%	55.9%		
	Student	405	272	99	98	469		
		46.30%	31.10%	11.30%	11.20%	53.7%		
	Retired	44	13	5	5	23		
		65.70%	19.40%	7.50%	7.50%	34.3%		
	Unemployed	20	22	10	5	37		
		35.10%	38.60%	17.50%	8.80%	64.9%		

The designation of “anxious” represents respondents who scored 5 to 21 in GAD-7 scale (i.e., mild, moderate, and severe anxiety); % within independent variable groups

### Prevalence depression

Table 3 shows that 40.42% of respondents scored within the normal range for depression levels (PHQ-9 = 0–4); 59.6% were considered to suffer from depression (PHQ-9 = 5–27). Table 3 also demonstrates that PA moderate and vigorous during the COVID-19 outbreak was significantly associated (*P* < 0.001) with depression. There was significant difference in the prevalence of depression between physically active and physically inactive people; there was also a significant difference in the prevalence of depression by gender (*P* < 0.001), and the prevalence of depression was significantly higher among college students (*P* < 0.003). Unlike anxiety, the prevalence of depression was significantly associated with marital status and occupation, as there was a significant difference in depression levels between married and single respondents, and between students and others (*P* < 0.001).

**Table 3:** Prevalence of depression (PHQ-9) among groups during the outbreak of COVID-19 in Kuwait

Sociodemographic characteristics		Depression level					Depressed	χ^2^	*P* value
		Normal	Mild	Moderate	Moderate to severe	Severe			
		519	399	199	108	59	765		
		40.42%	31.07%	15.50%	8.41%	4.60%	59.6%		
Physical activity	Physically inactive	222	222	132	72	45	471	57.694	< 0.001
		32.00%	32.00%	19.00%	10.40%	6.50%	68.0%		
	Physically active	297	177	67	36	14	294		
		50.30%	29.90%	11.30%	6.10%	2.40%	49.7%		
Gender	Female	176	176	102	81	42	401	84.697	< 0.001
		30.50%	30.50%	17.70%	14.00%	7.30%	69.5%		
	Male	343	223	97	27	17	364		
		48.50%	31.50%	13.70%	3.80%	2.40%	51.5%		
Education	High school and less	43	29	19	14	5	67	29.642	0.003
		39.10%	26.40%	17.30%	12.70%	4.50%	60.9%		
	Diploma	63	56	26	11	9	102		
		38.20%	33.90%	15.80%	6.70%	5.50%	61.8%		
	Bachelor’s degree	281	240	129	70	39	478		
		37.00%	31.60%	17.00%	9.20%	5.10%	63.0%		
	Higher education	132	74	25	13	6	118		
		52.80%	29.60%	10.00%	5.20%	2.40%	47.2%		
Marital status	Single	190	209	105	60	44	418	50.869	< 0.001
		31.30%	34.40%	17.30%	9.90%	7.20%	68.8%		
	Married	329	190	94	48	15	347		
		48.70%	28.10%	13.90%	7.10%	2.20%	51.3%		
Occupation	Employer	73	102	54	37	20	213	61.492	< 0.001
		25.50%	35.70%	18.90%	12.90%	7.00%	74.5%		
	Student	379	272	131	62	30	495		
		43.40%	31.10%	15.00%	7.10%	3.40%	56.6%		
	Retired	44	12	3	3	5	23		
		65.70%	17.90%	4.50%	4.50%	7.50%	34.3%		
	Unemployed	23	13	11	6	4	34		
		40.40%	22.80%	19.30%	10.50%	7.00%	59.6%		

“Depressed” column represents all respondents who scored 5 to 27 in PHQ-9 scale (i.e., mild, moderate, moderate to severe, and severe depression); % within independent variable groups

### Predictors of anxiety

Multiple regression models were performed for the continuous variables of anxiety score (*M* = 6.26 ± 5.5). A multicollinearity test was carried out to verify the existence of the association, and the result revealed that the VIF factor of the model was < 3, indicating no multicollinearity [10]. The residuals of both models were normally distributed [3] (see Appendix). The associations of potential predictors of anxiety during COVID-19 are presented in Table 4.

**Table 4:** Multiple regression results for anxiety

Anxiety	*B*	95% CI for *B*		SE B	β	*R2*	*AR2*
		LL	UL				
Model						0.078	0.074***
(Constant)	14.18***	12.323	16.042	0.948			
Gender	− 2.125***	− 2.719	− 1.531	0.30	− 0.192***		
Physical activity	− 1.507***	− 2.096	− 0.919	0.30	− 0.136***		
Education	− 0.493**	− .865-	− 0.121	0.19	− 0.072**		
Age	− .050-**	− 0.084	− 0.015	0.018	− 0.1**		
Marital status	0.381	− 0.343	1.104	0.369	0.035		
Occupation	0.045	− 0.436	0.527	0.246	0.006		

Note. *Model* “Enter” method in SPSS Statistics; *B* unstandardized regression coefficient; *CI* confidence interval; *LL* lower limit; *UL* upper limit; *SE B* standard error of the coefficient; *β* standardized coefficient; *R2* coefficient of determination; *AR2* adjusted R2; *p <> .05, **p < .01, ***p <. 001

A multiple regression was used to examine the relationship of anxiety with PA, gender, education, age, occupation, and/or marital status (Table 4). The regression model significantly predicted anxiety (*F*_(6,1277)_ = 18.028, *p* < 0.001, adj. *R*^2^ = 0.074, *R*^2^ = .279). PA, gender, education, and age added statistical significance to the prediction (*P < 0.05*)*.* The highest beta was for gender impact (*B* = 2.125), indicating that the women experienced greater anxiety during the COVID-19 outbreak than the men. The second highest beta was for the negative impact of PA (*B* = − 1.507), indicating that respondents engaged in more PA experienced lower anxiety during the COVID-19 outbreak. This finding was followed by the negative impact of education (*B* = − 0.493) indicating lower anxiety with higher education level. The least negative impact corresponded to age (*B* = − 0.05), indicating lower anxiety levels with older age. Marital status and occupation had no significant impact on anxiety during the COVID-19 outbreak in Kuwait (*P* > 0.05).

### Predictors of depression

Multiple regression models were performed for the continuous variables of depression score (*M* = 7.20 ± 5.9). A multicollinearity test was carried out to verify the existence of the association, and the result revealed that the VIF factor of the model was < 3, indicating no multicollinearity [10]. The residuals of both models were normally distributed [3] (see Appendix) The potential predictors of depression during COVID-19 are presented in Table 5.

**Table 5:** Multiple regression results for depression

Depression	*B*	95% CI for *B*		*SE B*	β	*R2*
		*LL*	*UL*			
Model						0.152
(Constant)	19.648***	17.75	21.546	0.97		
Gender	− 2.534***	− 3.140	− 1.927	0.31	− .215***	
Physical Activity	− 2.436***	− 3.036	− 1.835	0.31	− .207***	
Marital Status	− 0.965*	− 1.703	− 0.226	0.376	− 0.082	
Age	− .069***	− 0.104	− 0.034	0.018	− 0.13	
Occupation	0.249	− 0.741	0.243	0.251	− 0.028	
Education	− 0.244	− 0.624	0.136	0.194	− 0.034	

Note. *Model* “Enter” method in SPSS Statistics; *B* unstandardized regression coefficient; *CI* confidence interval; *LL* lower limit; *UL* upper limit; *SE B* standard error of the coefficient; *β* standardized coefficient; *R2* coefficient of determination; *AR2* adjusted R2; *p <> .05, **p < .01, ***p < .001

In addition, a multiple regression was used to examine the relationship of depression with PA, gender, education, age, occupation, and/or marital status (Table 5). The regression model significantly predicted depression (*F*_(6,1277)_ = 38.004, *P <* .001, adj. *R*^*2*^ *=* 0.152, *R*^2^ = 0.389). PA, gender, age, and marital status added statistical significance to the prediction, *P < 0.05*. The highest beta (*B*), at 2.534, was for gender impact, as women experienced higher levels of depression than men. The second highest beta was for the negative impact of PA (*B* = 2.436), indicating that respondents who engaged in higher levels of PA experienced lower levels of depression. This finding was followed by the impact of marital status (*B* = 0.965); married respondents indicated higher levels of depression than single respondents. Finally, the least negative impact was for age (*B* = 0.069), indicating that respondents older in age experienced the lowest levels of depression. Education and occupation variables had no significant impact on levels of depression among the population of Kuwait during the COVID-19 outbreak (*P* > 0.05).

## Discussion

This study examined the prevalence and predictors of anxiety and depression during the COVID-19 outbreak in Kuwait. The data showed that over half of the participants reported anxiety, while close to 60% reported depression during the COVID-19 outbreak. According to the results, symptoms of anxiety were more likely to appear in women and individuals with lower PA and education and who are younger while symptoms of depression were more prevalent among women, elderly, and individuals with lower level of PA and education and who are married. These findings confirm psychological effects of COVID-19 as the results from recent studies from China [15, 19, 31] and Australia [29]

In agreement with recent findings [9, 15], younger respondents showed greater levels of anxiety and depression during the COVID-19 outbreak in Kuwait. Although the reasons for this are complex, it may be due to that this segment of population were most exposed to news from social media, which is usually overwhelmed with fake news, unconfirmed information, and rumors [19, 21]. Additionally, suspending schools, colleges, and university in Kuwait might have imposed feelings of loneliness, boredom, uncertainty, and stress [26]. These negative feelings are usually associated adverse psychological implications including anxiety and depression.

In agreement with previous studies [13, 14, 31], and in contrast to others [15], women in the current study suffered from anxiety and depression at greater levels than men during the COVID-19 outbreak. Liu et al. [19] indicated that these psychological differences might be because women are more sensitive to psychological stress thus susceptible to experiencing anxiety and depression versus men [18, 31]. Greater prevalence of anxiety and depression might also be attributed to lower PA participation among women. Although Kuwait has experienced rapid social development, including more opportunities for women to exercise while maintaining privacy, cultural barriers often prevent women from sufficient PA participation. In accordance with previous research [1, 32], less women were physically active compared to men, although the difference was nonsignificant in the current study (Table 1). Furthermore, given the family responsibilities, women were most likely to stay home with the children during the 2-h gross period, which is an important outlet for stress relief.

More than 50% of the respondents in the current study reported no proper PA, which is similar to previously reported (57.23%) [1]. This might indicate that participation in PA was directly affected by COVID-19 outbreak. However, more studies are needed to verify these results and speculations. Numerous studies [4, 5] and meta-analyses [24, 30] have shown that exercise is associated with psychological benefits, including a reduction in the risk of anxiety and depression. The current findings confirmed that greater participation in PA is associated with lower anxiety and depression during the COVID-19 epidemic. The regression models showed that respondents who followed the standard PA guidelines during the COVID-19 outbreak reported normal levels of anxiety and depression (GAD-7 = 0–4; PHQ-9 = 0–4). This indicates the positive impact of PA during an epidemic and should serve to encourage people to become or remain active even during a total curfew. These findings suggest that the Kuwaiti government implementation of the 2-h grace period was beneficial in preventing more symptoms of anxiety and depression. Furthermore, this grace period should probably be extended a little longer for more PA engagement. Therefore, during future epidemics, stakeholders should respond rapidly and comprehensively to encourage widespread participation in PA, in or outside the home, to increase physical and mental health. These findings confirm an important point of view: While PA produces long-term benefits for mental health, it also produces immediate psychological benefits in terms of mood, anxiety, and stress [12, 27].

This is the first study to measure the prevalence of anxiety and depression during the pandemic in Kuwait. It is also the first study to examine the relationship between PA and anxiety and depression among the Kuwaiti population. Despite this, the study was limited by its cross-sectional nature, which challenges conclusions about long-term effects. It was also limited by the online survey, which may have prevented some people from participating (although, it should be noted, an online survey is one of few ways to reach people during total curfew measures). Additional limitation to be considered is the assessment of physical activity. A conventional PA questionnaire was not used in order to preserve the respondents’ time for the other questionnaires (i.e., PHQ-9 and GAD-7). The survey was conducted from 21 June to 11 July, and around that time over 158,000 expatriates have left Kuwait. This may explain the shortage of non-Kuwaiti respondents in this study.

## Conclusion

Consistent with hypothesis, findings of this study suggest that the COVID-19 outbreak may have greater psychological impact on women, younger and married individuals, and individuals with a lower level of education. However, regular PA appears to be an important long-term and immediate factor in reducing symptoms of anxiety and depression during an epidemic.

## Supplementary Information

**Additional file 1.** Appendix A.

## Data Availability

The datasets used and/or analyzed during the current study are available from the corresponding author on reasonable request.

## Abbreviations

PA: Physical activity
GAD-7: Generalized anxiety disorder
PHQ: Patient Health Questionnaire
PHQ-9: It is a module in PHQ which measures depression symptoms
PAAET: Public Authority for Applied Education and Training
