# Supplementary figures and images for: Anxiety and depression during the COVID-19 pandemic in Kuwait: the importance of physical activity
Source: Middle East Curr Psychiatry. 2020 Nov 3;27(1):60. doi: 10.1186/s43045-020-00065-6 (PMC7607368; doi:10.1186/s43045-020-00065-6)

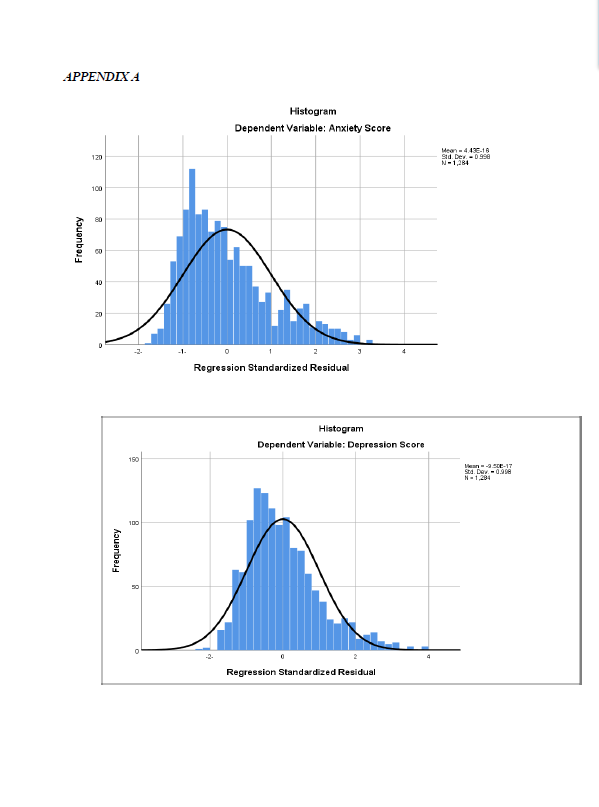

Supplement: Supplementary file 1 — Additional file 1. Appendix A. [file 43045_2020_65_MOESM1_ESM.png]
